# Daily process and key characteristics of phytoplankton bloom during a low-water level period in a large subtropical reservoir bay

**DOI:** 10.3389/fpls.2024.1390019

**Published:** 2024-04-16

**Authors:** Lu Tan, Lan Wang, Qinghua Cai

**Affiliations:** ^1^ State Key Laboratory of Freshwater Ecology and Biotechnology, Institute of Hydrobiology, Chinese Academy of Sciences, Wuhan, Hubei, China; ^2^ Hubei Key Laboratory of Wetland Evolution & Ecological Restoration, Wuhan Botanical Garden, Chinese Academy of Sciences, Wuhan, Hubei, China

**Keywords:** phytoplankton, daily sampling, VPA, db-RDA, β diversity partition, network analysis, TN/TP

## Abstract

Reservoirs, heavily influenced by artificial management, often harbor phytoplankton assemblages dominated by cyanobacteria or dinoflagellates, triggering significant changes in aquatic ecosystems. However, due to limited sampling frequency and insufficient attention to species composition, the bloom processes and key characteristics of phytoplankton community structure have not been systematically elucidated. During the low-water level period when blooms are most likely to occur (June to September) in a tributary bay of the Three Gorges Reservoir, daily sampling was conducted to investigate phytoplankton community composition, identify significant environmental factors, and evaluate important structure characteristics of phytoplankton community. The results showed that *Microcystis aeruginosa* maintained a clear dominance for almost a month in stage 1, with low Shannon and evenness but a high dominance index. Phytoplankton total density and biomass decreased drastically in stage 2, but *Microcystis aeruginosa* still accounted for some proportion. The highest Shannon and evenness but the lowest dominance index occurred in stage 3. *Peridiniopsis niei* occurred massively in stage 4, but its dominant advantages lasted only one to two days. NH_4_-N was responsible for the dominance of *Microcystis aeruginosa*, while TP and PO_4_-P was responsible for the dominance of *Peridiniopsis niei*; however, precipitation contributed to their drastic decrease or disappearance to some extent. The TN : TP ratio could be considered as an important indicator to determine whether *Microcystis aeruginosa* or *Peridiniopsis niei* dominated the phytoplankton community. Throughout the study period, physiochemical factors explained more variation in phytoplankton data than meteorological and hydrological factors. Pairwise comparisons revealed an increase in average β diversity with stage progression, with higher β diversities based on abundance data than those based on presence/absence data. Repl had a greater effect on β diversity differences based on presence/absence data, whereas RichDiff had a greater effect on β diversity differences based on species abundance data. Co-occurrence networks for stage 1 showed the most complex structure, followed by stage 4, while the network for stage 3 was relatively sparse, although the overall community division remained compact. This study provides a useful attempt to explore the status and changes in phytoplankton community structure during the bloom process through high-resolution investigation.

## 1 Introduction

Phytoplankton species are the most widespread and quantitatively relevant primary producers in aquatic ecosystems (Basset et al., 2008). A significant increase in phytoplankton biomass, known as a phytoplankton bloom, indicates an imbalance between phytoplankton growth and loss processes (Carstensen et al., 2007). It is widely accepted that global climate change and eutrophication have intensified phytoplankton blooms in lakes and reservoirs around the world (Ho et al., 2019; Griffith and Gobler, 2020; Liu et al, 2021). China has been severely threatened by water body eutrophication and frequent harmful phytoplankton bloom events during the past decade (Huang et al., 2020), which is one of the most challenging environmental problems (Huisman et al., 2018).

Reservoirs have become important frameworks within ecological studies of assemblage organization (Leitão et al., 2003), mainly due to the abiotic instability generated in their water bodies. Compared to lakes, reservoirs are subject to a greater degree of artificial management. Therefore, reservoirs can serve as a valuable water body type to study the ecological response of ecosystems to artificial interventions. Globally, reservoirs are inhabited by phytoplankton assemblages dominated by cyanobacteria such as *Microcystis* and *Dolichospermum*, or dinoflagellates such as *Ceratium*, forming either mixed or single blooms (see the literature cited by Bordet et al., 2017). Intense algal blooms can cause severe changes in the aquatic ecosystems (Reid et al., 2019), posing a significant threat to aquatic organisms and even humans using these water sources (Olokotum et al., 2020). Reservoir impoundment alters turbulence regimes, allowing the development of phytoplankton species that cannot thrive in fast-flowing waters, but gradually reach large populations in frequently stratified water columns with sufficient nutrients and light conditions (Bordet et al., 2017; Zeng et al., 2023). As the largest reservoir for water conservancy and hydropower projects in China, the Three Gorges Reservoir (TGR) has attracted widespread attention for its ecological impacts. Since the impoundment of the reservoir, the water level has risen significantly, the flow velocity has slowed down greatly, the water exchange has weakened, the artificial regulation has counteracted seasonal hydrological fluctuations (Ding et al., 2022; Ou et al., 2022), and the self-purification capacity of the water body has decreased (Liu et al., 2013). As a result, many tributary bays of the TGR have suffered from phytoplankton blooms of various sizes every year. In summer and early autumn (the low-water level period in the Three Gorges Reservoir), the light and water temperature conditions are good, which are favorable for algal growth, and algal blooms, especially cyanobacterial blooms, are prone to occur (Wang et al., 2011; Zhou et al., 2022). For example, a severe cyanobacterial bloom occurred in 2008 in Xiangxi Bay, a typical tributary of the Three Gorges Reservoir (Ministry of Environmental Protection of China, 2014), which seriously affected the safety of the water quality in the Three Gorges Reservoir area.

Research on phytoplankton bloom has primarily focused on the total amount of phytoplankton and the dominant species. However, the total algal abundance or biomass provides limited insight into the processes that govern their spatial and temporal distribution (Zhao et al., 2017). During blooms dominated by different species and at different stages of bloom development, the structure of phytoplankton communities can undergo significant changes with profound impacts on water quality. In addition, weekly sampling is essential for effective studies of phytoplankton blooms. This frequency is consistent with the timescale of population responses, as the generation time of phytoplankton varies from hours to a few days (Reynolds, 2006). While weekly dynamics of phytoplankton communities have been reported in Xiangxi Bay (Wang et al., 2011), changes in phytoplankton communities composition at higher sampling resolutions during the bloom process have not been documented. Therefore, more fine-scale and structured information on phytoplankton communities is urgently needed to fully understand the conceptual mechanisms driving bloom development.

The present study aimed to analyze phytoplankton communities during the bloom process using daily sampling data. Bloom stages were broadly defined based on total and relative abundance and biomass, as well as α diversity. We expected to identify differences in phytoplankton community structure within and between different bloom stages, and to explore the combined effects of environmental factors through a multifactorial investigation. The influence of environmental factors on the variation in phytoplankton community composition among sampling days (β diversity) was evaluated, and significant environmental factors were identified. In addition, β diversity was decomposed into turnover and richness changes to assess their relative importance. Co-occurrence networks were constructed to compare the internal structure of the phytoplankton community in different bloom stages.

## 2 Materials and methods

### 2.1 Study site and sampling

**Figure 1:**
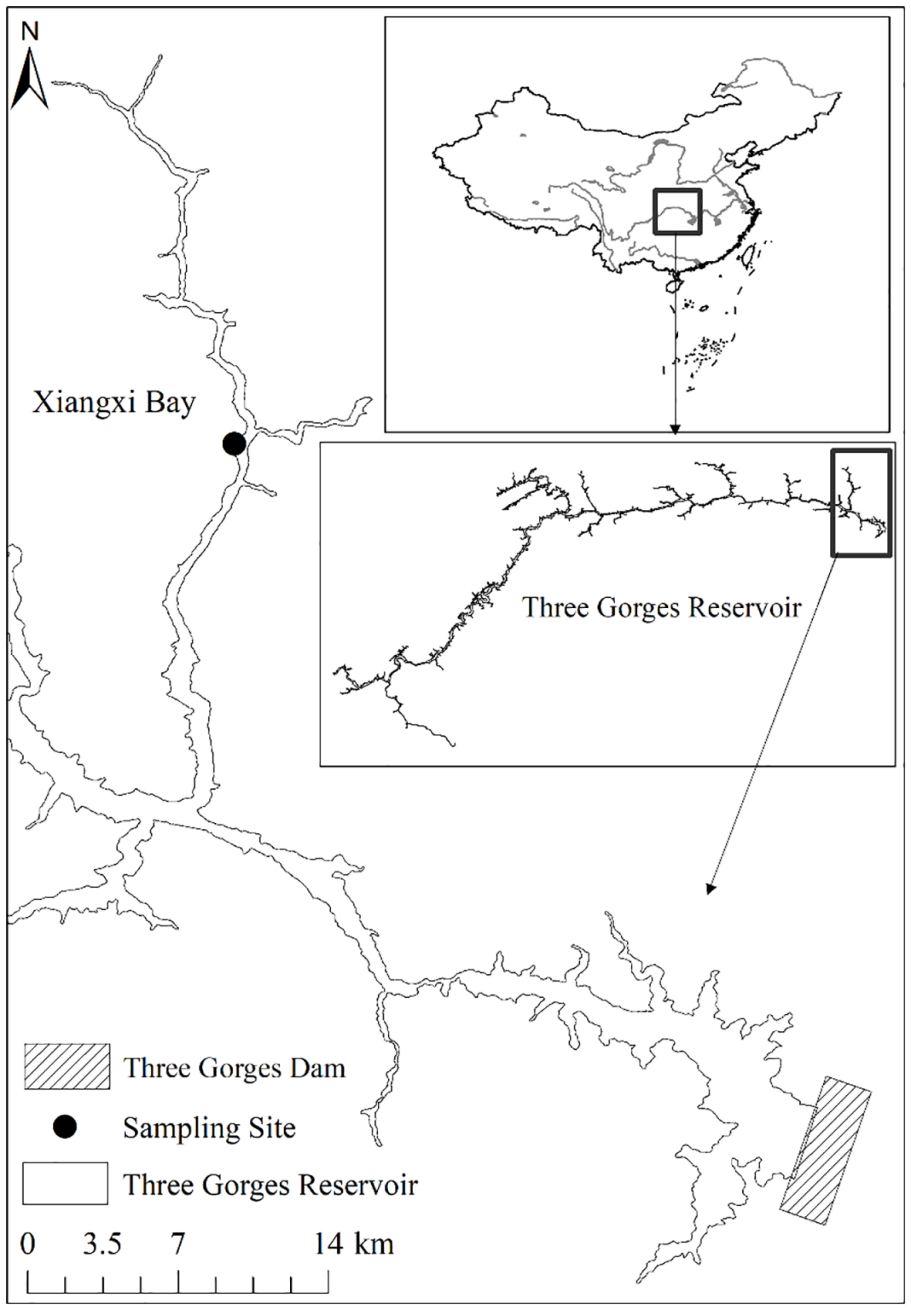
Location of the sampling site in Xiangxi Bay of the Three Gorges Reservoir, China.

The Three Gorges Reservoir (TGR), located at 29°16′~31°25′ N, 106°~110°50′ E, has a normal water level of 175 m above sea level and a summer flood protection level of 145 m, covers an area of 1080 km^2^ with a volumetric capacity of 3.93 × 10^10^ m^3^, and extends over 600 km in length and an average width of 1.1 km (Huang et al., 2006). A subtropical monsoon climate dominates this region (Cai et al., 2010), characterized by an average annual precipitation of 1000 to 1300 mm. As the largest tributary of the TGR in Hubei Province, the Xiangxi River is located in 38 km upstream of the Three Gorges Dam. It extends for 94 km as a mainstream and drains a watershed area of 3099 km^2^ (Wang et al., 1997). Notably, after the impoundment of the TGR, the lower 20~40 km stretch of the Xiangxi River was transformed into the Xiangxi Bay (Cai and Hu, 2006). 25 km upstream of the mouth of Xiangxi Bay, the sampling site in this study was located at the Xiangxi Ecosystem Station of the Institute of Hydrobiology, Chinese Academy of Sciences and China Three Gorges Corporation (**Figure 1**).

Daily sampling was conducted between June 18^th^ and September 28^th^, 2008, during a warm flood season characterized by small water level fluctuations around the low water level, i.e. 145 m a.s.l. Water samples were consistently collected at 10: 00 a.m., at a depth of 0.5 m below the water surface using a 5 L Van Dorn sampler. For nutrient analysis, samples were carefully stored in pre-cleaned plastic bottles and immediately acidified with sulfuric acid. Phytoplankton samples were preserved with neutral Lugol’s solution immediately after collection.

### 2.2 Abiotic and biotic variable measurements and acquisition

Daily precipitation (Prep), vertical profiles of water temperature, and wind speed (Wind) were recorded using EcoTech monitoring stations (EcoTech Umwelt-Meßsysteme GmbH, Germany). Photosynthetic active radiation (PAR) in the air and vertical profiles of PAR through the water column were obtained using a quantum sensor (Li-192SA, USA). Real-time water level data of the TGR were obtained from China Three Gorges Corporation. In this study, the water level (WL) data were derived from the daily average water level, while the diurnal fluctuation of water level (DFWL) was calculated based on the daily fluctuation range of water level.

Surface water temperature (WT), conductivity (Cond), dissolved oxygen (DO), and pH were measured using environmental monitoring systems (YSI 6600EDS, USA). Total phosphorus (TP), total nitrogen (TN), phosphate phosphorus (PO_4_-P), ammonia nitrogen (NH_4_-N), nitrate nitrogen (NO_3_-N), and dissolved silicon (DSi), were analyzed using a Continuous flow analyzer (Skalar San^++^, Netherlands) according to the Standard Observation and Measurement Protocol for Aquatic Ecosystems of the Chinese Ecosystem Research Network (CERN) (Cai, 2007).

A sedimentation method was used prior to phytoplankton analysis (Cai, 2007). Taxonomic identification of phytoplankton species followed the procedures described by Hu and Wei (2006) and John, Whitton and Brook (2002). Algal enumeration was performed using Fuchs-Rosental slides and an Olympus CX21 model microscope (Olympus Corporation, Japan) at 400× magnification.

### 2.3 Data analysis

The Relative Water Column Stability (RWCS) indicates the thermal stratification conditions within a water body based on density variations throughout the water column (Padisák et al., 2003). This dimensionless parameter was calculated by comparing the density gradient across the water column with the difference in density of pure water at 4°C and 5°C, according to the formula described by Padisák et al. (2003):


$$RWCS=\frac{D_{b}-D_{S}}{D_{4}-D_{5}}$$

where D_b_, D_s_, D_4_, and D_5_ represent the densities of the bottom water, surface water, and pure water at 4°C and 5°C, respectively. In this study, a depth of 1 m was considered the “surface layer”, while the maximum depth was considered the “bottom layer” (with a water depth of approximately 10 m). The euphotic zone (Zeu) was defined as the depth at which 1% of the photosynthetically active radiation penetrates the surface (Naselli-Flores, 2000). Transparency, as indicated by the Secchi depth (SD), was measured using a Secchi disk.

Algal biomass was assessed using formulas applicable to geometric shapes, assuming the fresh weight unit expressed in mass, where 1 mm^3^/L is equal to 1 mg/L (Wetzel and Likens, 2000). The Shannon index (Shannon), Pielou’s evenness index (Evenness), and Dominance index (Dominance) were used to characterize α diversity. The Shannon index takes into account both the number of individuals and the number of taxa and is calculated using the formula:


$$\text{S}\text{h}\text{a}\text{n}\text{n}\text{o}\text{n}\;=\;-\text{s}\text{u}\text{m}(\left({\text{n}}_{\text{i}}/\text{n}\right)\text{l}\text{n}({\text{n}}_{\text{i}}/\text{n}))$$

where n_i_ is the number of individuals of taxon i and n is the total number of individuals. Evenness is determined by dividing Shannon diversity by the logarithm of the number of taxa. Dominance is calculated as:


$$\;\text{D}\text{o}\text{m}\text{i}\text{n}\text{a}\text{n}\text{c}\text{e}\;=\;-\text{s}\text{u}\text{m}{\left({\text{n}}_{\text{i}}/\text{n}\right)}^{2}\;\;\;\;\;\;\;\;\;$$

The Compositional dissimilarities of phytoplankton communities between different days (β diversity) were analyzed using both presence/absence data (using Podani family, Jaccard-based indices) and abundance data (using the Ruzicka index) in the R package “adespatial”. This analysis allowed for the partitioning of dissimilarities into replacement and richness difference components, providing insight into the turnover and richness changes within the community over time. Correlation-based co-occurrence network analysis was used to explore the co-occurrence patterns among phytoplankton taxa. Pairwise Spearman’s rank correlations (r) were calculated using the R package “psych”. Only correlations with r greater than 0.8 or less than -0.8 and statistically significant with a *p*-value threshold of less than 0.01 were included in the network analyses. Network visualization and modular analysis were performed using Gephi (version 0.9.2). Topological characteristics including modularity, clustering coefficient, average degree, and average path length were calculated to provide quantitative insights into the structure and organization of the network.

Friedman’s two-way analysis of variance by ranks was used to compare differences between phytoplankton bloom stages in abiotic and biotic parameters. Linear correlation between environmental variables was assessed using Pearson’s correlation coefficient. To investigate the relationship between phytoplankton community (total density, total biomass, density of *Microcystis aeruginosa* and *Peridiniopsis niei*, and α diversity index Shannon, evenness, and dominance) and environmental variables, we performed a Mantel test using the R package “LinkET”. The Variation Partitioning Analysisi (VPA) was performed based on the partitioning criterion of meteorological and hydrological (Prep, Wind, PAR, WL, DFWL, and RWCS), physical (Zeu, SD, WT, Cond), and chemical (DO, pH, TN, NH4-N, NO3-N, TP, PO4-P, DSi, and TN/TP) factors partition criterion. Distance-based redundancy analysis (db-RDA) is an ordination method similar to redundancy analysis (RDA), with the difference that non-Euclidean dissimilarity indices, such as the Bray-Curtis distance, can be considered (Oksanen et al., 2022). db-RDA successfully partitions data variability based on complex designs or models, and applies an appropriate multivariate distance measure for ecological datasets, by computing principal coordinates and adjusting for negative eigenvalues when necessary, with a constant added to squared distances (Mcardle and Anderson, 2001). In this study, db-RDA was performed to explore relationships between phytoplankton species composition and environmental factors during daily processes using the R package “vegan”.

## 3 Results

### 3.1 Daily phytoplankton bloom process

**Figure 2:**
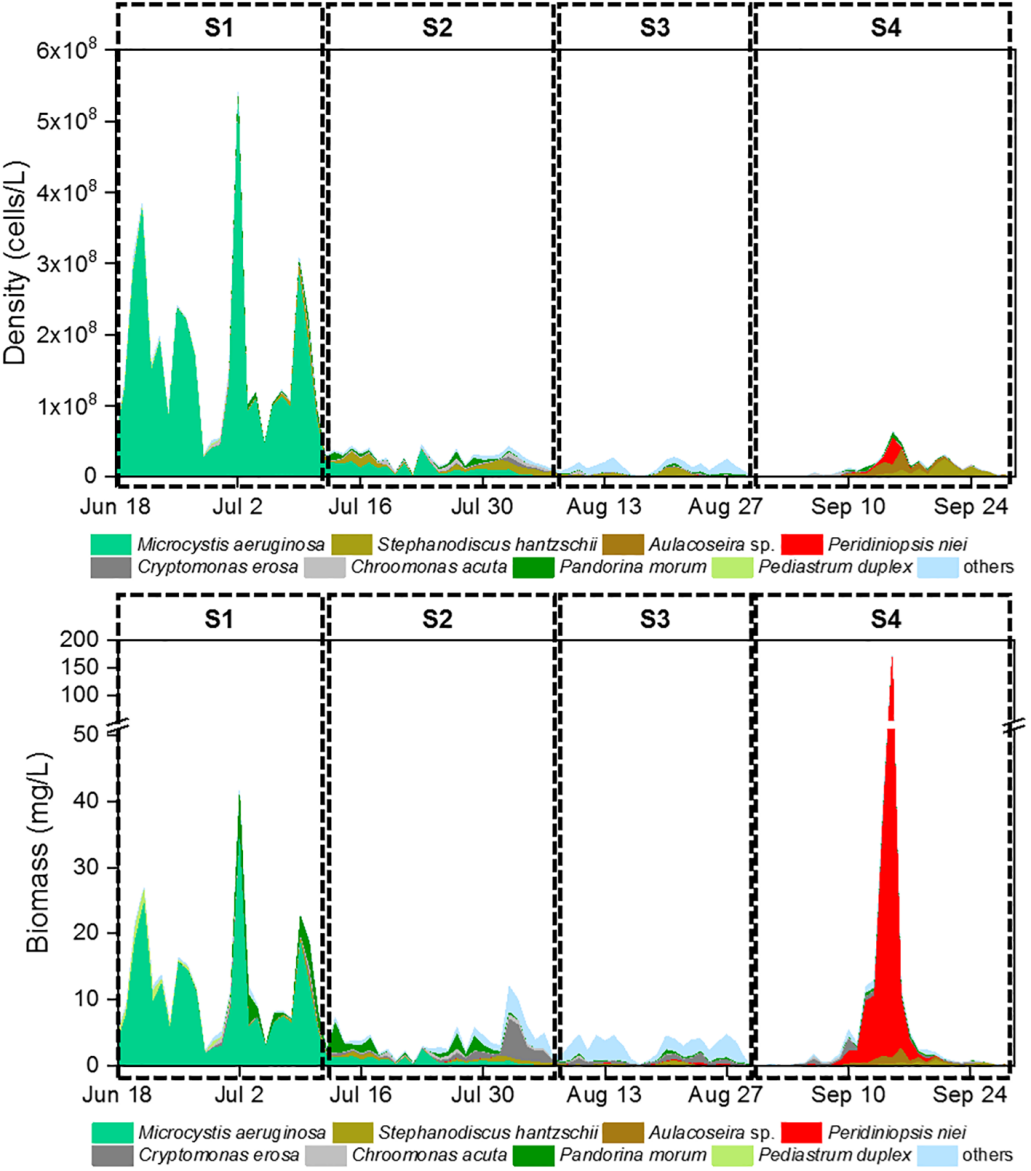
Daily changes of phytoplankton density and phytoplankton biomass.

**Figure 3:**
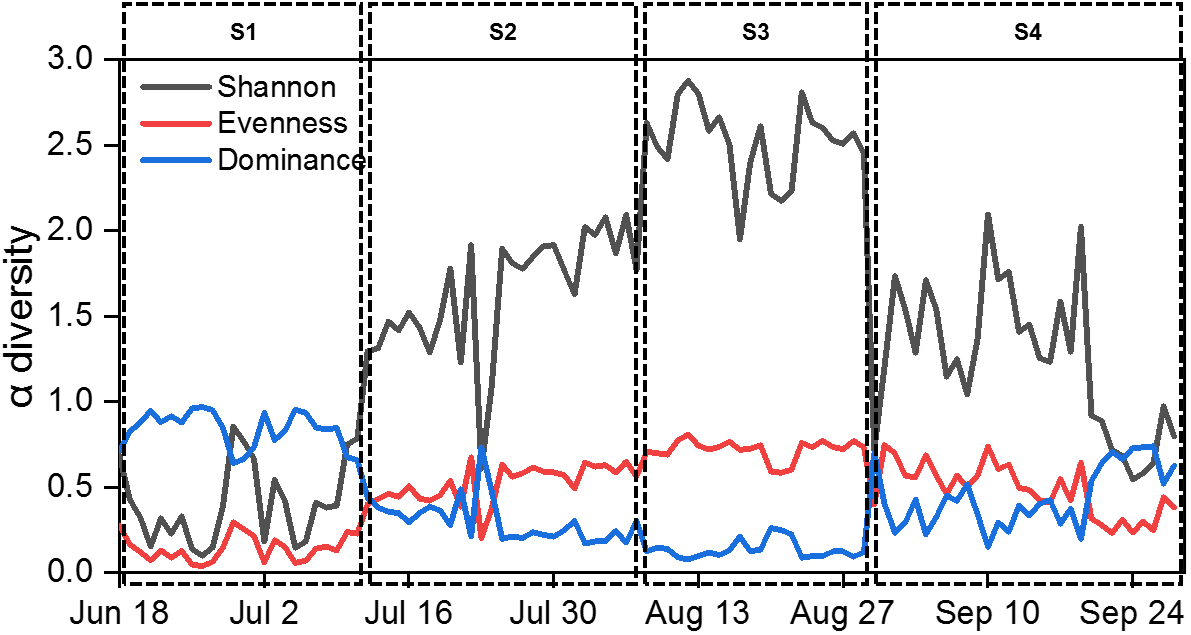
Daily changes of α diversity indices during the study period.

Euglenophyta (3 species). Daily changes in density and biomass are shown in **Figure 2**. *Microcystis aeruginosa* (Maer) and *Peridiniopsis niei* (Pnie) emerged as the most significant species when both density and biomass were considered simultaneously. *Microcystis aeruginosa* maintained a clear dominance for almost a month, with both its relative density and biomass exceeding 80% on most days. On July 2^nd^, its density and biomass peaked at 5.23E8 cells/L and 34.22 mg/L, respectively. *Peridiniopsis niei* briefly dominated in mid-September. On September 15^th^, its density and biomass peaked at 3.79E7 cells/L and 167.89 mg/L, respectively, representing 59.1% of the total density and 98.1% of the total biomass on that day. The α diversity indices Evenness, Shannon, and dominance showed clear temporal patterns (**Figure 3**). Evenness and Shannon followed a similar trend, with Shannon showing a wide range of variation. Both indices showed low values during the bloom periods of *Microcystis aeruginosa* and *Peridiniopsis niei*. Shannon reached its highest values around August and the second highest values from mid-July to early August. In contrast, Dominance showed opposite changes and was higher during the blooms of *Microcystis aeruginosa* and *Peridiniopsis niei*.

Based on the daily variation of density, biomass and α diversity indices, the study period was divided into four stages, designated S1 to S4 (**Table 1**). During S1, spanning from June 18^th^ to July 11^th^, the stage was characterized by a bloom of *Microcystis aeruginosa* which dominated the community. This stage had the lowest Shannon and evenness and the highest dominance. S2, occurring from July 12^nd^ to August 7^th^, represented a stage in which the dominance of *Microcystis aeruginosa* decreased, and other species including *Peridiniopsis niei* began to appear. S3, from August 8^th^ to 29^th^, was characterized by a continued decrease in total density and a reduction of total biomass to the minimal levels. *Peridiniopsis niei* continued to increase, and the highest Shannon and evenness and the lowest dominance was recorded. S4, from August 30^th^ to September 28^th^, represented the stage of *Peridiniopsis niei* bloom. During this stage, the total density decreased to the minimum level, while the total biomass showed drastic variations. A statistical analysis was performed on all environmental factors and the aforementioned biotic factors to evaluate their average values, ranges of variation among these four stages, and the significance of the differences using Friedman’s two-way analysis of variance by ranks in **Table 1**. The results showed significant differences between the stages for all factors except the meteorological factors.

**Table 1:** Statistical summary of environmental conditions, phytoplankton density, biomass and diversity.

	S1 (Jun 18~Jul 11)	S2 (Jul 12~Aug 7)	S3 (Aug 8~29)	S4 (Aug 30~Sep 28)	Differences
Prep (mm/day)	5.50 (0.00~37.00)	4.86 (0.00~36.20)	10.72 (0.00~77.60)	1.83 (0.00~36.10)	0.851
Wind (m/s)	1.51 (0.40~3.40)	1.54 (0.50~3.70)	1.55 (0.30~4.20)	1.25 (0.10~3.90)	0.315
PAR (mol/m^2^/day)	34.29 (9.89~59.39)	36.80 (8.06~52.39)	31.43 (3.78~50.98)	23.93 (5.86~44.23)	0.148
WL (m)	145.50 (145.02~145.88)	145.57 (145.21~145.88)	145.75 (145.31~145.90)	145.73 (145.21~145.87)	<0.001
DFWL (m/day)	0.23 (0.09~0.70)	0.24 (0.09~0.44)	0.19 (0.08~0.51)	0.17 (0.06~0.51)	0.002
RWCS	64.75 (37.76~108.52)	94.71 (41.55~166.82)	89.88 (52.05~174.22)	57.59 (18.32~122.28)	<0.001
Zeu (m)	2.88 (1.76~4.67)	3.96 (2.63~5.03)	3.22 (2.05~3.87)	3.21 (0.57~4.34)	<0.001
SD (m)	0.97 (0.20~1.80)	1.18 (0.80~2.00)	0.92 (0.40~1.20)	0.87 (0.10~1.50)	0.001
WT (°C)	26.02 (24.87~27.45)	27.73 (26.04~28.70)	27.06 (24.92~29.02)	24.46 (19.83~26.51)	<0.001
Cond (μs/cm)	321.46 (293.00~343.00)	302.89 (277.00~319.00)	273.73 (165.00~328.00)	217.70 (102.00~307.00)	<0.001
DO (mg/L)	13.51 (9.65~17.50)	10.80 (6.94~13.46)	10.90 (7.04~15.96)	9.78 (7.01~14.22)	0.001
pH	8.95 (8.60~9.20)	8.57 (8.10~9.00)	8.60 (8.10~9.00)	8.56 (8.00~9.20)	<0.001
TN (mg/L)	1.53 (1.14~2.30)	1.41 (1.08~2.09)	1.26 (1.07~1.69)	1.19 (0.79~2.45)	<0.001
NH_4_-N (mg/L)	0.28 (0.02~0.74)	0.12 (0.02~0.29)	0.08 (0.01~0.21)	0.06 (0.01~0.29)	<0.001
NO_3_-N (mg/L)	1.09 (0.69~1.43)	1.16 (0.87~1.46)	1.06 (0.88~1.43)	1.02 (0.68~2.32)	0.002
TP (mg/L)	0.06 (0.02~0.13)	0.04 (0.01~0.09)	0.03 (0.01~0.06)	0.09 (0.01~0.30)	<0.001
PO_4_-P (mg/L)	0.04 (0.01~0.09)	0.02 (0.01~0.05)	0.02 (0.01~0.06)	0.04 (0.01~0.20)	<0.001
DSi (mg/L)	2.94 (2.43~3.53)	3.07 (2.30~3.79)	3.37 (2.47~4.37)	6.36 (4.93~8.48)	<0.001
TN/TP	30.16 (11.62~61.00)	45.57 (16.67~145.00)	48.88 (19.50~113.00)	19.77 (4.30~49.00)	<0.001
Density (cells/L)	1.68E8 (3.00E7~5.41E8)	2.75E7 (2.72E6~4.50E7)	1.51E7 (7.80E5~2.79E7)	1.35E7 (1.58E5~6.42E7)	<0.001
Biomass (mg/L)	12.59 (2.15~41.60)	3.97 (0.45~12.01)	3.03 (0.22~4.75)	9.29 (0.03~171.10)	<0.001
Maer density (cells/L)	1.56E8 (2.76E7~5.23E8)	1.14E7 (1.03E6~3.83E7)	2.87E5 (0~2.24E6)	2.11E3 (0~6.32E4)	<0.001
Pnie density (cells/L)	0	1.17E3 (0~1.05E4)	2.16E4 (0~1.37E5)	1.83E6 (0~3.79E7)	<0.001
Shannon	0.41 (0.10~0.86)	1.64 (0.61~2.09)	2.52 (1.95~2.88)	1.24 (0.55~2.10)	<0.001
Evenness	0.15 (0.04~0.30)	0.52 (0.20~0.68)	0.72 (0.59~0.81)	0.48 (0.23~0.75)	<0.001
Dominance	0.84 (0.64~0.97)	0.31 (0.17~0.73)	0.14 (0.08~0.26)	0.45 (0.15~0.74)	<0.001

### 3.2 Response of phytoplankton community to environmental factors

**Figure 4:**
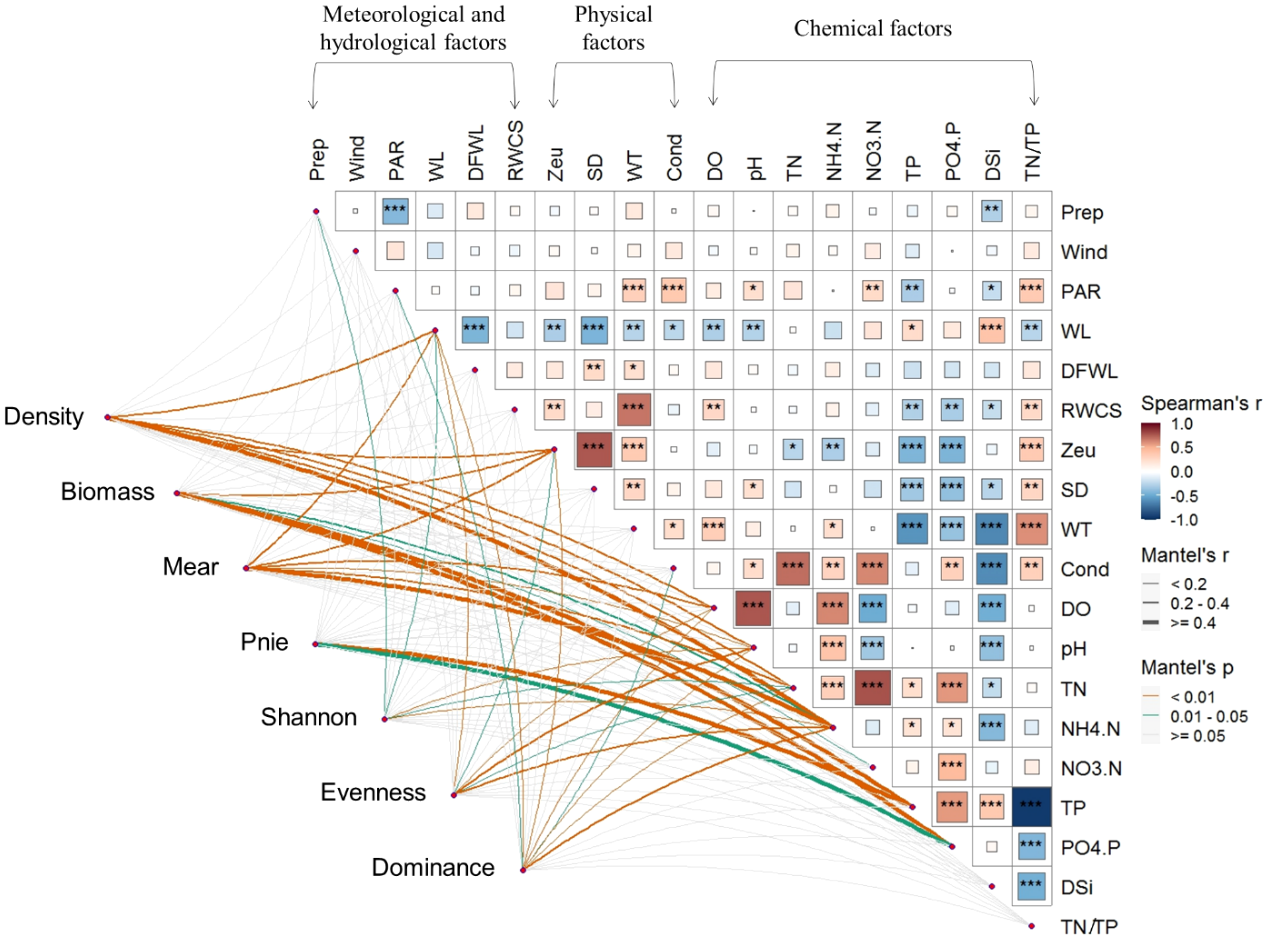
Relationship between environmental variables and phytoplankton total density (density), total biomass (biomass), density of Maer (Maer) and Pnie (Pnie), α diversity indices (Shannon, Evenness, and Dominance) through Mantel tests. Pairwise comparisons of different environmental variables are presented in the top-right section.*0.01≤p<0.05, **0.001≤p<0.01, *** denotes p<0.001

Pairwise comparisons of different environmental variables in **Figure 4** revealed stronger correlations between physical and chemical factors, as well as between internal chemical factors. Significantly strong positive correlations (Spearman’s r > 0.6) were observed between SD and Zeu, WT and RWCS, pH and DO, NH_4_-N and DO, TN/TP and WT, and pairwise between TN, NO_3_-N and Cond. Significantly strong negative correlations (Spearman’s r< -0.6) were found only between DSi and WT, DSi and Cond, and TN/TP and TP. Phytoplankton total density and *Microcystis aeruginosa* density showed a stronger correlation with NH_4_-N. Total phytoplankton biomass and the density of *Peridiniopsis niei* showed a stronger correlation with TP and PO_4_-P. Evenness showed a relatively higher correlation with pH and NH_4_-N, while dominance showed a relatively higher correlation with NH_4_-N.

**Figure 5:**
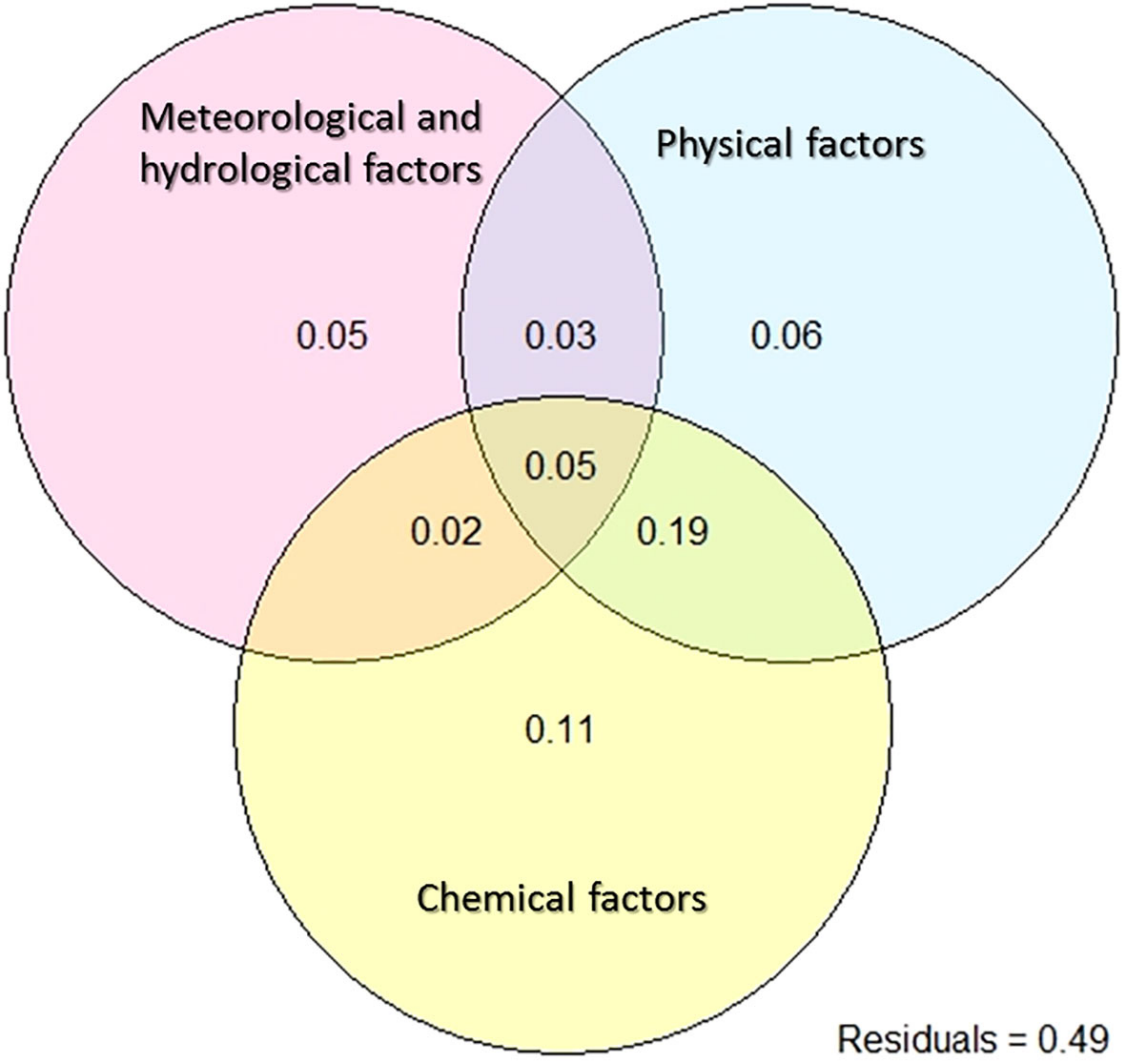
Variation partitioning analysis to show the percentages of explained variations for the phytoplankton composition. Variation was partitioned by meteorological and hydrological factors (Prep, Wind, PAR, WL, DFWL, and RWCS), physical factors (Zeu, SD, WT, and Cond) and chemical factors (DO, pH, TN, NH_4_-N, NO_3_-N, TP, PO_4_-P, DSi, TN/TP).

Many explanatory variables closely associated with the phytoplankton community were collected in this study, but their collective effect on changes within the phytoplankton community remains elusive. In addition, there are individual effects and interactions among these explanatory variables that pique our interest. Variation partitioning analyses (VPA) decompose total variability into the effects of pure explanatory variables, interaction effects between explanatory variables, and unexplained residuals, and help to address the aforementioned questions. In this study, phytoplankton variation was explained by 19 environmental factors, categorized into three groups: six meteorological and hydrological factors, four physical factors, and nine chemical factors. Together, they accounted for 0.51 of the total variation (**Figure 5**). Individually, their explanatory power was 0.05, 0.06, and 0.11, respectively. The interaction effect between the three groups was 0.05. Notably, the interaction between physical and chemical factors emerged as the most significant explanation for the observed variation in phytoplankton community composition, contributing 0.19 to the total variation.

**Figure 6:**
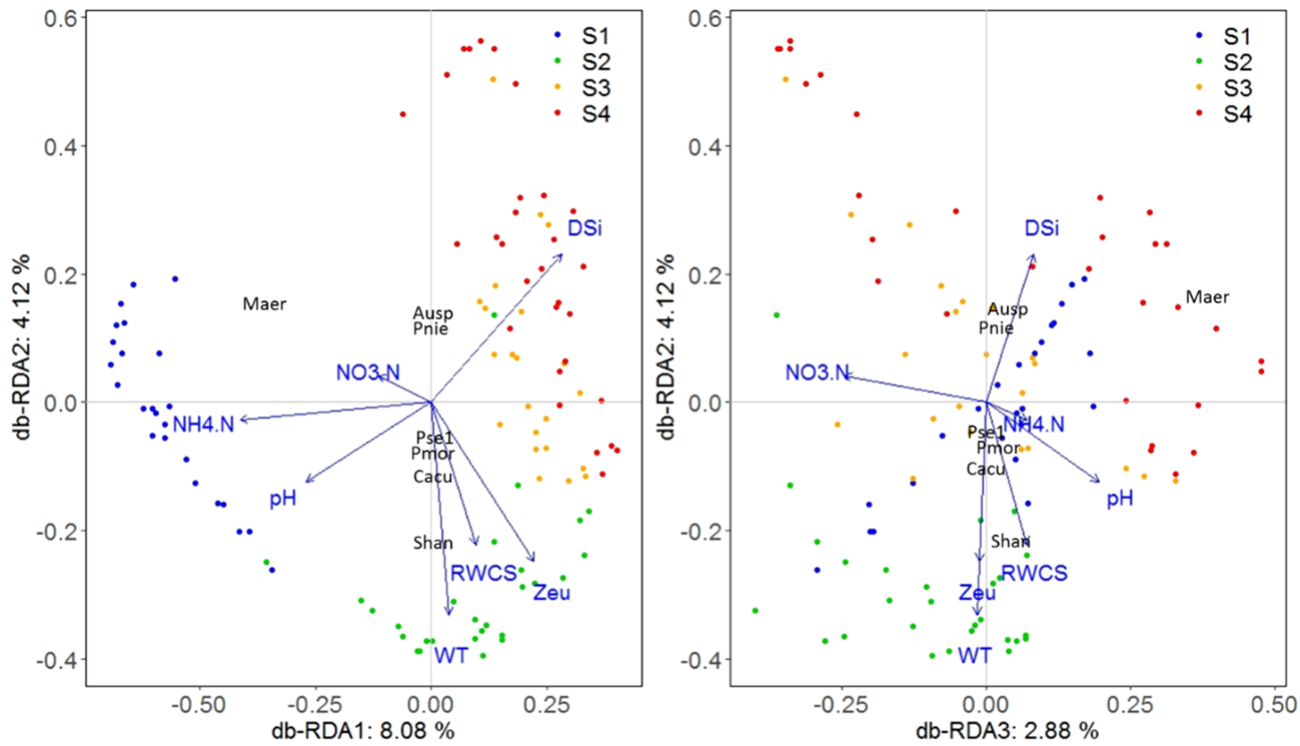
Phytoplankton community ordination based on db-RDA technique.

The db-RDA technique was used to investigate the dissimilarity (Bray-Curtis distance) between phytoplankton communities among different sampling days and their relationships with environmental factors (**Figure 6**). Forward selection was used to identify significant environmental factors, including DSi, NO_3_-N, NH_4_-N, pH, WT, RWCS, and Zeu in this study. Variance Inflation Factor (VIF) testing indicated no significant collinearity among the environmental factors (VIF< 4.5). Collectively, all db-RDA axes explained 18.82% of the total variability in phytoplankton data, with the first three axes contributing 8.08%, 4.12%, and 2.88%, respectively. Key environmental factors associated with the first axis were DSi (0.18), NH_4_-N (-0.26), and pH (-0.17); with WT (-0.21), Zeu (-0.15), and RWCS (-0.14) for the second axis; and with NO_3_-N (-0.15) for the third axis. Global tests showed significance for all db-RDA axes under the reduced model (F = 4.3781, p = 0.001, 999 permutations). Individual tests showed significance for each of the first three axes (F = 13.1637, p = 0.007; F = 6.7134, p = 0.007; F = 4.6818, p = 0.007). There was a clear separation of phytoplankton communities among the four stages. Stage S1 sites had higher NH_4_-N and pH with *Microcystis aeruginosa* as the dominant species. Stage S2 sites had higher WT, Zeu, and RWCS, with *Stephanodiscus hantzschii* and *Chroomonas acuta* as dominant species. Stage S4 sites had higher DSi, with *Peridiniopsis niei* and *Aulacoseira* sp. as dominant species.

### 3.3 Structure characteristics of phytoplankton community

**Figure 7:**
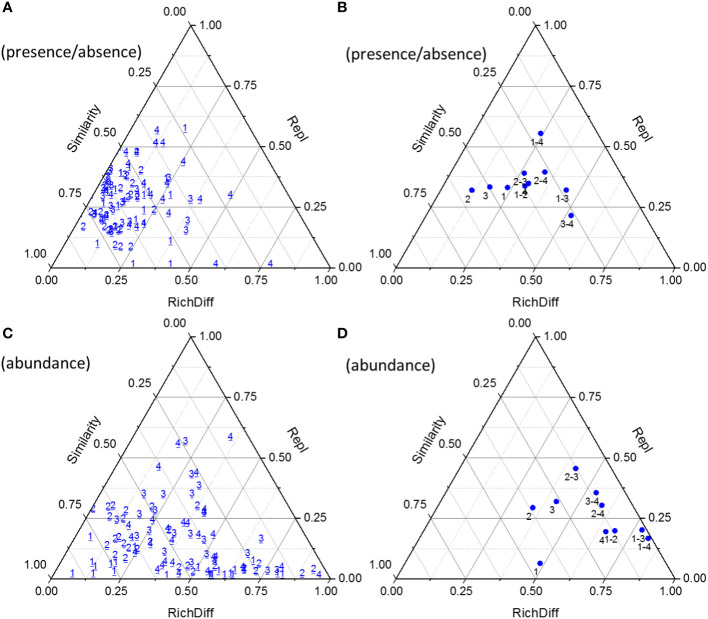
Triangular plots of β diversity comparisons for phytoplankton communities between successive days (with presence/absence data, **(A)** with abundance data, **(C)** and between days within and between stages (with presence/absence data, **(B)** with abundance data, **(D)**. The position of each point is determined by a triplet of values from the Similarity, RichDiff (richness difference), and Repl (replacement) matrices; each triplet sums to 1. The numbers 1~4 indicated the stage 1~4.

The β diversity of the phytoplankton community was assessed and further partitioned into richness difference (RichDiff) and replacement (Repl) in **Figure 7**. Lower similarity in **Figure 7** indicated higher β diversity, and vice versa. The β diversities based on abundance data were higher than those based on presence/absence data. Pairwise comparison of phytoplankton communities in stage 4 showed the lowest average similarity and the highest β diversity. In all stages, Repl contributed more to the variation than RichDiff with presence/absence data, while RichDiff contributed more to the variation than Repl with abundance data. Pairwise comparisons revealed an increase in average β diversity with stage progression, i.e., β diversity in stage 1< stages 1-2< 1-3< 1-4, 2< 2-3< 2-4, and 3< 3-4. For presence/absence data, RichDiff contributed more between stages 1 and 3, and stages 3 and 4, while Repl contributed more between the other pairs of stages. For abundance data, Repl contributed more between stages 2 and 3, while RichDiff contributed more between the other pairs of stages.

**Figure 8:**
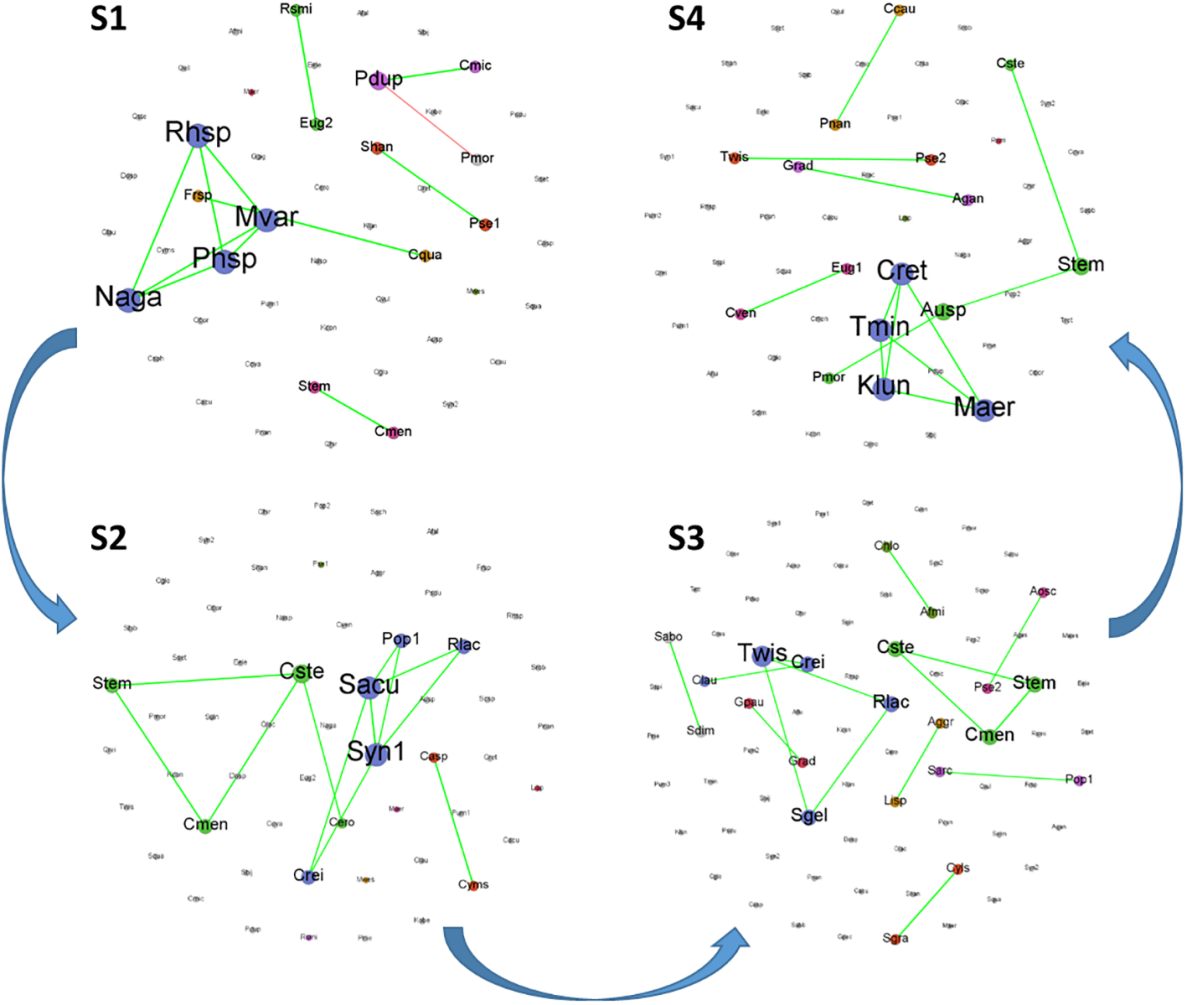
Co-occurrence networks of phytoplankton community at species level based on Spearman correlation analysis for each stage. (Green and red curves between nodes represent positive and negative correlations, respectively).

The co-occurrence network was used to elucidate the dynamic interactions and structure within the phytoplankton communities over time for each stage (**Figure 8**). In each stage, the proportion of species forming the network was relatively low, with most species changing independently. Among the species forming the network, there were some shared species across stages 2, 3, and 4, for example, *Stephanodiscus minutulus* and *Cyclotella stelligera*. However, in stage 1, the only shared species was *Pandorina morum*. The Topological characteristics of the co-occurrence networks for each stage are presented in **Table 2**. All nodes (i.e., all species) were shown to facilitate comparison of differences between the stages, but only those edges with connections were shown. Stages 1 and 3 showed almost opposite topological characteristics. Stage 1 had the highest average degree, graph density, and average clustering coefficient, and the lowest connected components and average path length. However, stage 3 had the lowest average degree, average weighted degree, graph density, and the highest modularity, connected component, and average path length. In addition, S2 had the lowest modularity, and S4 had the lowest average clustering coefficient.

**Table 2:** Topological characteristics of co-occurrence networks for each stage.

	S1	S2	S3	S4
Nodes	49	57	77	57
Edges	12	12	15	13
Average Degree	0.49	0.421	0.39	0.456
Average Weighted Degree	0.401	0.38	0.347	0.436
Graph Density	0.01	0.008	0.005	0.008
Modularity	0.668	0.557	0.824	0.699
Connected Components	40	49	64	47
Average Clustering Coefficient	0.8	0.792	0.762	0.667
Average Path Length	1.077	1.294	1.35	1.25

## 4 Discussion

Algal blooms have emerged as a significant environmental problem in inland waters (Hou et al., 2022) and pose a significant threat to public health and aquatic ecosystems worldwide (Brooks et al., 2016). Daily sampling provides a more detailed view of the phytoplankton bloom process compared to the broader dynamics captured by weekly sampling. Our previous weekly study (Wang et al., 2011) missed the peaks of both *Microcystis aeruginosa* on July 2^nd^ and *Peridiniopsis niei* on September 15^th^, despite overlapping study periods. Inaccurate time scales can lead to misinterpretation of key factors influencing algal community composition and confound studies of growth dynamics (Gupta et al., 2023). There is an urgent need for fine-scale and structured information on phytoplankton communities to fully understand the conceptual mechanisms driving bloom development, which will provide technical support for watershed management. Different studies use different definitions of the biomass threshold for identifying bloom occurrence. For example, Tett (1987) defines exceptional blooms as those that exceed a chlorophyll threshold of 100 µg/L. In practice, a chlorophyll *a* level of 15 µg/L is often used as a standard for algaecide application (Hudnell et al., 2010). In our study, the peak chlorophyll *a* levels in stages 1 and 4 reached 70.80 µg/L and 706.44 µg/L, respectively (unpublished data). However, it is not appropriate to compare two types of phytoplankton blooms, *Microcystis* and *Peridiniopsis* blooms, based on chlorophyll *a* concentrations alone. The *Microcystis* bloom had higher density but lower biomass and chlorophyll *a* concentration and lasted longer. In contrast, the *Peridiniopsis* bloom had lower density but higher biomass and chlorophyll *a* concentration, with its clear advantage lasting one to two days. Consideration of phytoplankton community structure provides a more insightful framework for studying bloom dynamics.

Species dominance in the environment is typically not attributed to the influence of a single environmental factor, but is often determined by complex nonlinear relationships involving many environmental variables. Wind- driven hydrodynamic conditions are often an important factor in determining the extent of phytoplankton blooms (Wu et al., 2015). Light and the hydrodynamic conditions also play a critical role in the development and maintenance of blooms (Wang et al., 2020; Summers and Ryder, 2023). However, in this study, the influence of meteorological and hydrological factors was less than that of physiochemical factors (**Figure 5**). Excessive nutrient enrichment in water bodies undoubtedly promotes the growth of harmful cyanobacteria (Nowicka-Krawczyk et al., 2022). Throughout the study period, the density of *Microcystis aeruginosa* showed a stronger correlation with NH_4_-N (**Figure 4**). The *Microcystis* bloom created conditions that could further increase its dominance, such as higher pH and lower Zeu (**Table 1**). These conditions favor the development of cyanobacterial blooms (Amorim and Moura, 2021). In addition to being photoautotrophic, cyanobacteria are known to use several ecophysiological strategies to outcompete eukaryotic algae and ensure their mass dominance in the phytoplankton. These strategies include the presence of gas vesicle aggregates, rapid cell division, and the use of specific metabolic pathways, among others (Li et al., 2016). Summers and Ryder (2023) concluded that prolonged stratification reduces competition, decreases diversity, and leads to cyanobacteria-dominated blooms. In our study, stages 1 and 2 had relatively higher RWCS, but there was a drastic decrease in the abundance of *Microcystis*, due in part to reductions in TN and NH_4_-N. This suggests that nutrient availability may contribute more significantly than stratification to the transformation of the phytoplankton community towards a reduction of *Microcystis* blooms. Furthermore, the influence of rainfall could not be excluded, as rainfall occurred on July 1^st^ (11.6 mm/day) and 2^nd^ (0.3 mm/day).

Freshwater dinoflagellates, including *Peridiniopsis*, are found in many aquatic ecosystems worldwide and can sometimes lead to harmful blooms that adversely affect water quality (Viner-Mozzini et al., 2003; Yatigammana et al., 2011). Dinoflagellates possess several abilities, such as tolerance to high irradiance, vertical migration within the water column to optimize photosynthesis and growth, and the secretion of toxins, which allow them to compete, thrive, and dominate within the phytoplankton community (Viner-Mozzini et al., 2003; Regel et al., 2004). In our study, *Peridiniopsis niei* density showed a stronger correlation with TP and PO_4_-P, highlighting the importance of phosphorus in maintaining their dominance. Wu et al. (2017) also showed that high phosphorus increased the photosynthetic efficiency of *Peridiniopsis* and stimulated its growth. However, certain environmental factors, such as lower RWCS in stage 4, did not provide suitable conditions to maintain their competitive advantage. The dominance of *Peridiniopsis niei* persisted for one to two days, possibly due to the rainfall events that occurred on September 15^th^ (0.1 mm/day). A similar phenomenon was reported by Zhou et al. (2011), who attributed the decrease in phytoplankton biomass and the disappearance of blooms to heavy rainfall.

**Figure 9:**
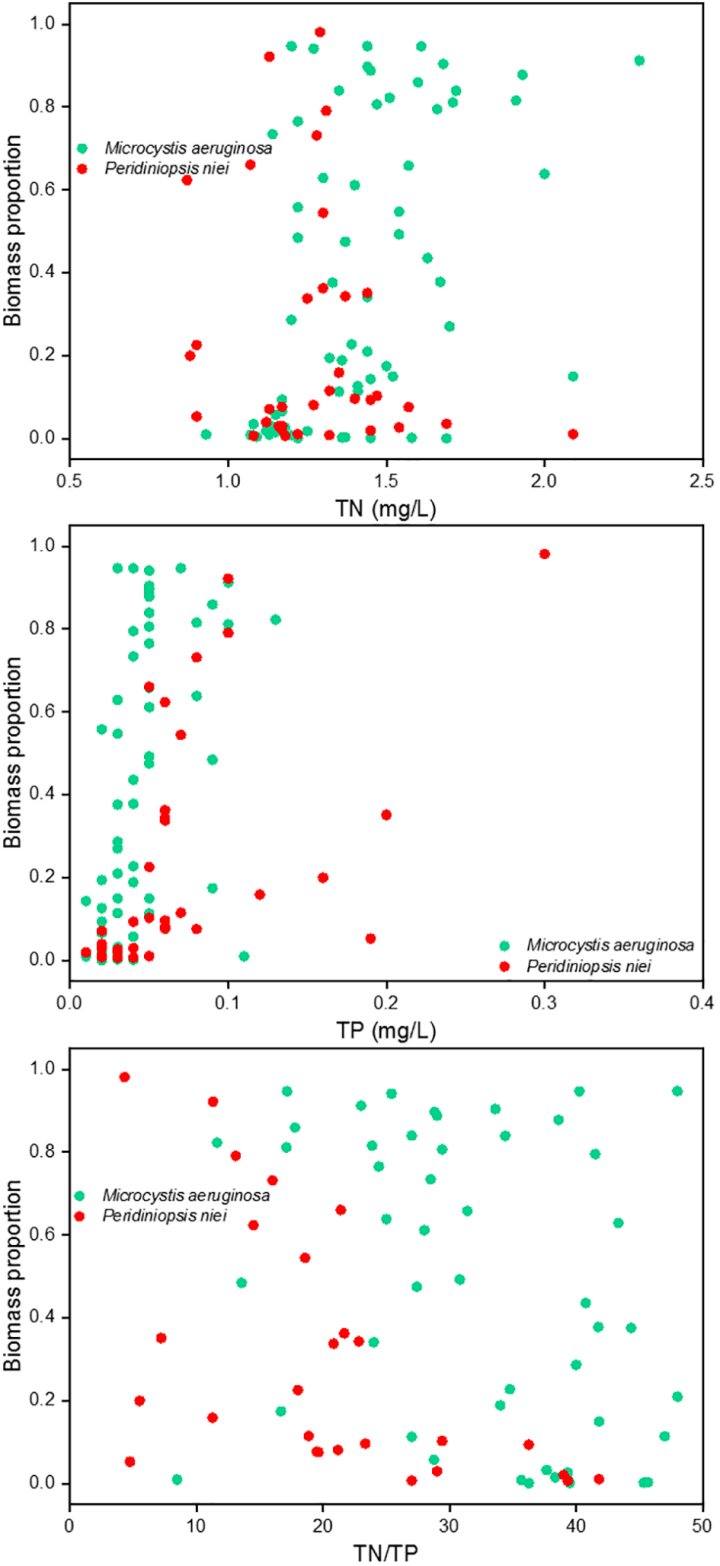
Correlation relationships between TN, TP, as well as TN : TP and biomass proportion of *Microcystis aeruginosa* and *Peridiniopsis niei*.

Despite the morphological and physiological differences between cyanobacteria and dinoflagellates, reservoirs provide ecological conditions conducive to their success. The possession of aerotopes or flagella allows individuals to dominate in newly created regimes with less turbulence (Bordet et al., 2017). Although TP reduction is widely used as a primary strategy to mitigate the severity of harmful algal blooms (Pick, 2016), cases have shown that TP decreases can be accompanied by increases in chlorophyll *a* concentrations, suggesting that other abiotic factors may also influence the observed trends (Huang et al., 2020). The study by Nowicka-Krawczyk et al. (2022) showed that the composition of cyanobacterial blooms is strongly influenced by the nitrogen/phosphorus (N/P) ratio, with N/P explaining 13.3% of the variation and PO_4_-P explaining 10.5%. In hypereutrophic waters (TP > 0.1 mg/L), phytoplankton biomass was found to increase with additions of both phosphorus and nitrogen (Filstrup and Downing, 2017). Therefore, it is critical to consider the TN : TP ratio for these systems (Summers and Ryder, 2023). Some studies have considered N/P as a factor influencing phytoplankton structure, rather than just absolute N and P concentrations (Liu et al., 2024; Yang et al., 2020). In this study, the maximum concentrations of TP reached 0.13 and 0.30 mg/L in stages 1 and 4, respectively. Therefore, the ratio of TN : TP should be considered. We correlated TN, TP, and TN : TP with the biomass proportion of *Microcystis aeruginosa* and *Peridiniopsis niei* for the sampling days when either *Microcystis aeruginosa* or *Peridiniopsis niei* was present (see **Figure 9**). The results showed that a high TN concentration, low TP concentration, and high TN : TP ratio corresponded to a high biomass proportion of *Microcystis aeruginosa*. Conversely, a low TN concentration, high TP concentration, and low TN : TP ratio corresponded to a high biomass proportion of *Peridiniopsis niei*. This contrasting response of *Microcystis aeruginosa* and *Peridiniopsis niei* suggests that TN : TP plays an important role in maintaining the competitive advantages of different dominant bloom species.

The bloom-forming taxa significantly influenced the composition and structure of the phytoplankton. *Microcystis aeruginosa* blooms significantly reduced phytoplankton species richness, Shannon diversity, and evenness, reaching the lowest levels observed (refer to **Figure 3** and **Table 2**), consistent with the findings of Escalas et al. (2019). Similarly, blooms of *Peridiniopsis niei* also resulted in reduced phytoplankton species richness, Shannon diversity, and evenness, consistent with recent findings by Amorim and Moura (2021). In addition, intense blooms of cyanobacteria and dinophyta are known to cause fish kills by depleting oxygen concentrations during biomass decomposition (Meichtry de Zaburlín et al., 2016), leading to significant disruptions in aquatic food webs, ecosystem functioning, and ecosystem service provision (Sukenik et al., 2015). However, throughout the entire study period, stage 3 exhibited low total density and total biomass, with the highest species richness (as indicated by the highest nodes in **Table 2**), Shannon diversity, and evenness, as well as the lowest dominance index. The successful growth of *Peridiniopsis niei* after stage 3, despite the initial low phytoplankton density, indicates its resilience under reduced competition from other phytoplankton.

The β diversity of phytoplankton can serve as an excellent model for elucidating the mechanisms that shape aquatic communities in reservoirs (de Moura et al., 2022). Studies examining replacement rates and variations in phytoplankton richness or abundance can be interpreted as indicators of ecosystem function, given the significant dependence of reservoir trophic webs on phytoplankton dynamics (Znachor et al., 2020). In this study, β diversities based on abundance data were higher than those based on presence/absence data. Repl had a greater effect on β diversity differences based on presence/absence data. This may indicate that during blooms dominated by different species and at different stages of bloom development, certain species had replaced others, resulting in variations in β diversity. Conversely, RichDiff had a greater effect on β diversity differences based on species abundance data. This suggests the presence of species with significant differences in abundance between samples, particularly dominant phytoplankton bloom species, and that these differences in abundance were one of the main drivers of β diversity differences. These results indicate that β diversity differences throughout the phytoplankton bloom process included significant species turnover and abundance gradients, both of which jointly influenced the differences in phytoplankton species composition on different sampling days. Szabo et al. (2019) suggested that both abundance and binary data sets should be considered when assessing ecological uniqueness, as they can shed light on different ecological interpretation patterns, and de Moura et al. (2022) also identified some differences between data types.

Increasing evidence suggests that the ability of microbial systems to support ecological functions and resist environmental pressures is an emergent property resulting from interactions among many taxa (Escalas et al., 2019). Network-based approaches provide an integrated representation of interactions among different taxa within microbial communities (Deng et al., 2012). However, studies on the structure of phytoplankton networks are still limited (Carey et al., 2017; Tarafdar et al., 2023). Each phytoplankton network constructed in this study represents the covariation in phytoplankton density over time for each stage, with similar responses to environmental changes or interactions among phytoplankton species (Rottjers and Faust, 2018). The co-occurrence networks of stages 1 and 4 had relatively high average degree and graph density, low connected components and average path length. This combination of features may reflect a relatively dense and evenly connected network, where connections between nodes are tight and the entire network is a relatively cohesive unit. Such a network structure may indicate efficient information propagation, shorter path lengths, and higher network accessibility under certain circumstances. Although composed of fewer nodes and links for stage 1 in **Table 2**, the *Microcystis*-dominated networks could be considered as more complex than the others, as they have a higher graph density and a higher average number of links (degree) per node. Similar results were reported in the study of Escalas et al. (2019). Furthermore, the co-occurrence networks in stage 3 were relatively special, with the lowest average degree and graph density, but the highest number of nodes, edges, modularity, connected components, and average path length. This indicated that there were some sparsely connected small-world structures within the community, resulting in a lower clustering coefficient for nodes within the community; however, the overall community division remained compact. It has been reported that networks with small-world structures tend to be more robust to perturbations and random loss of species (Peura et al., 2015). A slightly more positive correlation at stage 3 also suggests that high cooperative interactions among species may result in a more stable community structure and thus more resilient to environmental changes (Tarafdar et al., 2023).

## 5 Conclusion

The study analyzed the dynamics of the phytoplankton community during a low-water level period (June to September) when blooms of *Microcystis aeruginosa* and *Peridiniopsis niei* occurred sequentially in a large subtropical reservoir bay. The entire bloom process was clearly delineated into four stages based on daily changes in total and relative phytoplankton abundance and biomass, and α diversity including Shannon, evenness and dominance. *Microcystis aeruginosa* maintained significant dominance for almost a month in stage 1, with low Shannon and evenness but a high dominance index. This was followed by stage 2, where total density and biomass decreased dramatically, but *Microcystis aeruginosa* still accounted for some proportion. Phytoplankton total density and biomass continued to decrease in stage 3, which was characterized by the highest Shannon and evenness but the lowest dominance index. *Peridiniopsis niei* bloomed in stage 4, but its dominant advantages lasted only one to two days. Total phytoplankton density and *Microcystis aeruginosa* density showed a stronger correlation with NH_4_-N, while total phytoplankton biomass and *Peridiniopsis niei* density showed a stronger correlation with TP and PO_4_-P. The TN : TP ratio could be considered as an important indicator to determine whether *Microcystis aeruginosa* or *Peridiniopsis niei* dominated the phytoplankton community. Precipitation partially contributed to their significant decrease or disappearance. Variation partitioning analyses (VPA) indicated that physical and chemical factors and their interactions explained more variation in phytoplankton data than meteorological and hydrological factors. DSi, NH_4_-N, pH, WT, Zeu, and RWCS were identified as key environmental factors associated with phytoplankton community variation using the db-RDA technique. The β diversity of the phytoplankton community was assessed and further partitioned into richness difference (RichDiff) and replacement (Repl). The results showed an increase in average β diversity with stage progression, i.e., β diversity in stage 1< stages 1-2< 1-3< 1-4, 2< 2-3< 2-4, and 3< 3-4. In all stages, Repl contributed more to the variation than RichDiff with presence/absence data, while RichDiff contributed more to the variation than Repl with abundance data. Co-occurrence network analysis revealed that stage 1 had the highest average degree, graph density, and average clustering coefficient, along with the lowest connected components and average path length, indicating the most complex structure. However, stage 3 had the lowest average degree, average weighted degree, graph density, along with the highest modularity, connected component, and average path length, suggesting that the network for stage 3 was relatively sparse, despite maintaining a compact overall community division.

## Data availability statement

The raw data supporting the conclusions of this article will be made available by the authors, without undue reservation.

## Author contributions

LT: Data curation, Investigation, Writing – original draft. LW: Formal analysis, Methodology, Visualization, Writing – review & editing. QC: Conceptualization, Project administration, Writing – review & editing.

## References

[B1] Amorim C. A. Moura A. N. (2021). Ecological impacts of freshwater algal blooms on water quality, plankton biodiversity, structure, and ecosystem functioning. Sci. Total Environ. 758, 143605. doi: 10.1016/j.scitotenv.2020.143605 33248793

[B2] Basset A. Carrada G. C. Fedele M. Sabetta L. (2008). Equilibrium concept in phytoplankton communities. Encyclopedia Ecology Acad. Press 2, 1394–1402. doi: 10.1016/B978-008045405-4.00711-4

[B3] Bordet F. Fontanarrosa M. S. O'Farrell I. (2017). Influence of light and mixing regime on bloom - forming phytoplankton in a subtropical reservoir. River Res. Appl. 33, 1315–1326. doi: 10.1002/rra.3189

[B4] Brooks B. W. Lazorchak J. M. Howard M. D. Johnson M. V. V. Morton S. L. Perkins D. A.. (2016). Are harmful algal blooms becoming the greatest inland water quality threat to public health and aquatic ecosystems? Environ. Toxicol. Chem. 35, 6–13. doi: 10.1002/etc.3220 26771345

[B5] Cai Q. (2007). Protocols for standard observation and measurement in aquatic ecosystems (Beijing, China: Chinese Environmental Science Press).

[B6] Cai Q. Hu Z. (2006). Studies on eutrophication problem and control strategy in the Three Gorges Reservoir. Acta Hydrobiologica Sin. 30, 7–11. doi: 10.3321/j.issn:1000-3207.2006.01.002

[B7] Cai Q. Liu M. He Y. Zeng X. Jiang T. (2010). Climate change impact assessment report for the three gorges reservoir area of the yangtze river (Beijing, China: China Meteorological Press).

[B8] Carey C. C. Brown B. L. Cottingham K. L. (2017). The cyanobacterium *Gloeotrichia echinulata* increases the stability and network complexity of phytoplankton communities. Ecosphere 8, e01830. doi: 10.1002/ecs2.1830

[B9] Carstensen J. Henriksen P. Heiskanen A. S. (2007). Summer algal blooms in shallow estuaries: Definition, mechanisms, and link to eutrophication. Limnology Oceanography 52, 370–384. doi: 10.4319/lo.2007.52.1.0370

[B10] de Moura W. B. da Silva P. R. L. Baumgartner G. Bueno N. C. Bortolini J. C. (2022). Site contributions to phytoplankton beta diversity along two subtropical reservoirs. Aquat. Sci. 84, 59. doi: 10.1007/s00027-022-00890-3

[B11] Deng Y. Jiang Y. Yang Y. He Z. Luo F. Zhou J. (2012). Molecular ecological network analyses. BMC Bioinf. 13, 1–20. doi: 10.1186/1471-2105-13-113 PMC3428680 22646978

[B12] Ding D. Arif M. Liu M. Li J. Hu X. Geng Q.. (2022). Plant-soil interactions and C: N: P stoichiometric homeostasis of plant organs in riparian plantation. Front. Plant Sci. 13. doi: 10.3389/fpls.2022.979023 PMC9376457 35979078

[B13] Escalas A. Catherine A. Maloufi S. Cellamare M. Hamlaoui S. Yéprémian C.. (2019). Drivers and ecological consequences of dominance in periurban phytoplankton communities using networks approaches. Water Res. 163, 114893. doi: 10.1016/j.watres.2019.114893 31351356

[B14] Filstrup C. Downing J. (2017). Relationship of chlorophyll to phosphorus and nitrogen in nutrient-rich lakes. Inland Waters 7, 385–400. doi: 10.1080/20442041.2017.1375176

[B15] Griffith A. W. Gobler C. J. (2020). Harmful algal blooms: a climate change co-stressor in marine and freshwater ecosystems. Harmful Algae 91, 101590. doi: 10.1016/j.hal.2019.03.008 32057338

[B16] Gupta A. Hantush M. M. Govindaraju R. S. (2023). Sub-monthly time scale forecasting of harmful algal blooms intensity in Lake Erie using remote sensing and machine learning. Sci. Total Environ. 900, 165781. doi: 10.1016/j.scitotenv.2023.165781 37499836 PMC10552934

[B17] Ho J. C. Michalak A. M. Pahlevan N. (2019). Widespread global increase in intense lake phytoplankton blooms since the 1980s. Nature 574, 667–670. doi: 10.1038/s41586-019-1648-7 31610543

[B18] Hou X. Feng L. Dai Y. Hu C. Gibson L. Tang J.. (2022). Global mapping reveals increase in lacustrine algal blooms over the past decade. Nat. Geosci. 15, 130–134. doi: 10.1038/s41561-021-00887-x

[B19] Hu H. Wei Y. (2006). The freshwater algae of China—Systematics, taxonomy and ecology (Beijing, China: Science Press).

[B21] Huang Z. Li Y. Chen Y. Li J. Xing Z. Ye M.. (2006). Water quality prediction and water environmental carrying capacity calculation for Three Gorges Reservoir (Beijing, China: China WaterPower Press).

[B20] Huang J. Zhang Y. Arhonditsis G. B. Gao J. Chen Q. Peng J. (2020). The magnitude and drivers of harmful algal blooms in China’s lakes and reservoirs: A national-scale characterization. Water Res. 181, 115902. doi: 10.1016/j.watres.2020.115902 32505885

[B22] Hudnell H. K. Jones C. Labisi B. Lucero V. Hill D. R. Eilers J. (2010). Freshwater harmful algal bloom (FHAB) suppression with solar powered circulation (SPC). Harmful Algae 9, 208–217. doi: 10.1016/j.hal.2009.10.003

[B23] Huisman J. Codd G. A. Paerl H. W. Ibelings B. W. Verspagen J. M. H. Visser P. M. (2018). Cyanobacterial blooms. Nat. Rev. Microbiol. 16, 471–483. doi: 10.1038/s41579-018-0040-1 29946124

[B24] Leitão M. Morata S. M. Rodriguez S. Vergon J. P. (2003). The Effect of perturbations on phytoplankton assemblages in a deep reservoir (Vouglans, France). Hydrobiologia 502, 73–83. doi: 10.1023/B:HYDR.0000004271.08002.73

[B25] Li X. Dreher T. Li R. (2016). An overview of diversity, occurrence, genetics and toxin production of bloom-forming *Dolichospermum* (Anabaena) species. Harmful Algae 54, 54–68. doi: 10.1016/j.hal.2015.10.015 28073482

[B26] Liu D. Huang Y. Ji D. (2013). Algal blooms and ecological regulation in tributaries of Three Gorges Reservoir China (Beijing, China: Water Resources and Hydropower Press).

[B28] Liu Q. Wang Y. Li Y. Li Y. Wang Y. Zhou B.. (2021). Nutrient alteration drives the impacts of seawater acidification on the bloom-forming dinoflagellate *karenia mikimotoi* . Front. Plant Sci. 12. doi: 10.3389/fpls.2021.739159 PMC8572056 34751224

[B27] Liu F. Zhang H. Wang Y. Yu J. He Y. Wang D. (2024). Hysteresis analysis reveals how phytoplankton assemblage shifts with the nutrient dynamics during and between precipitation patterns. Water Res. 251, 121099. doi: 10.1016/j.watres.2023.121099 38184914

[B29] Mcardle B. H. Anderson M. J. (2001). Fitting multivariate models to community data: A comment on distance-based redundancy analysis. Ecology 82, 290–297. doi: 10.1890/0012-9658(2001)082[0290:FMMTCD]2.0.CO;2

[B30] Meichtry de Zaburlín N. Vogler R. E. Molina M. J. Llano V. M. (2016). Potential distribution of the invasive freshwater dinoflagellate *Ceratium furcoides* (Levander) Langhans (Dinophyta) in South America. J. Phycology 52, 200–208. doi: 10.1111/jpy.12382 27037585

[B31] Ministry of Environmental Protection of China (2014). Bulletin on the ecological and environmental monitoring results of the TGP, (2003-2013). Available online at: https://www.cnemc.cn/zzjj/jgsz/sts/gzdt_sts/

[B32] Naselli-Flores L. (2000). Phytoplankton assemblages in twenty-one Sicilian reservoirs: relationships between species composition and environmental factors. In Reynolds C.S. Dokulil M. Padisák J. (eds) The Trophic Spectrum Revisited. Developments in Hydrobiology, 150 (Dordrecht, Netherlands: Springer).

[B33] Nowicka-Krawczyk P. Żelazna-Wieczorek J. Skrobek I. Ziułkiewicz M. Adamski M. Kaminski A.. (2022). Persistent cyanobacteria blooms in artificial water bodies—an effect of environmental conditions or the result of anthropogenic change. Int. J. Environ. Res. Public Health 19, 6990. doi: 10.3390/ijerph19126990 35742239 PMC9223187

[B34] Oksanen J. Simpson G. L. Blanchet F. G. Kindt R. Legendre P. Minchin P. R.. (2022). Package ‘vegan’: Community Ecology Package. R package version 2.6-4. Available online at: https://rdrr.io/cran/vegan/

[B35] Olokotum M. Mitroi V. Troussellier M. Semyalo R. Bernard C. Montuelle B.. (2020). A review of the socioecological causes and consequences of cyanobacterial blooms in Lake Victoria. Harmful Algae 96, 101829. doi: 10.1016/j.hal.2020.101829 32560832

[B36] Ou T. Zhang M. Huang Y. Wang L. Wang F. Wang R.. (2022). Role of rhizospheric Bacillus megaterium HGS7 in maintaining mulberry growth under extremely abiotic stress in hydro-fluctuation belt of three gorges reservoir. Front. Plant Sci. 13. doi: 10.3389/fpls.2022.880125 PMC9195505 35712602

[B37] Padisák J. Barbosa F. Koschel R. Krienitz L. (2003). Deep layer cyanoprokaryota maxima in temperate and tropical lakes. Archiv Für Hydrobiologie (Special Issues Advanced Limnology) 58, 175–199.

[B38] Peura S. Bertilsson S. Jones R. I. Eiler A. (2015). Resistant microbial cooccurrence patterns inferred by network topology. Appl. Environ. Microbiol. 81, 2090–2097. doi: 10.1128/AEM.03660-14 25576616 PMC4345367

[B39] Pick F. R. (2016). Blooming algae: a Canadian perspective on the rise of toxic cyanobacteria. Can. J. Fisheries Aquat. Sci. 73, 1149–1158. doi: 10.1139/cjfas-2015-0470

[B40] Regel R. H. Brookes J. D. Ganf G. G. (2004). Vertical migration, entrainment and photosynthesis of the freshwater dinoflagellate *Peridinium cinctum* in a shallow urban lake. J. Plankton Res. 26, 143–157. doi: 10.1093/plankt/fbh008

[B41] Reid A. J. Carlson A. K. Creed I. F. Eliason E. J. Gell P. A. Johnson P. T. J.. (2019). Emerging threats and persistent conservation challenges for freshwater biodiversity. Biol. Rev. 94, 849–873. doi: 10.1111/brv.12480 30467930

[B42] Reynolds C. S. (2006). Ecology of phytoplankton (Cambridge, England: Cambridge University Press).

[B43] Rottjers L. Faust K. (2018). From hairballs to hypotheses-biological insights from microbial networks. FEMS Microbiol. Rev. 42, 761–780. doi: 10.1093/femsre/fuy030 30085090 PMC6199531

[B44] Sukenik A. Quesada A. Salmaso N. (2015). Global expansion of toxic and non-toxic cyanobacteria: effect on ecosystem functioning. Biodiversity Conserv. 24, 889–908. doi: 10.1007/s10531-015-0905-9

[B45] Summers E. J. Ryder J. L. (2023). A critical review of operational strategies for the management of harmful algal blooms (HABs) in inland reservoirs. J. Environ. Manage. 330, 117141. doi: 10.1016/j.jenvman.2022.117141 36603251

[B46] Szabo B. Lengyel E. Padisak J. Kovacs C. S. (2019). Benthic diatom metacommunity across small freshwater lakes: driving mechanisms, β-diversity and ecological uniqueness. Hydrobiologia 828, 183–198. doi: 10.1007/s10750-018-3811-9

[B47] Tarafdar L. Mohapatra M. Muduli P. R. Kumar A. Mishra D. R. Rastogi G. (2023). Co-occurrence patterns and environmental factors associated with rapid onset of *Microcystis aeruginosa* bloom in a tropical coastal lagoon. J. Environ. Manage. 325, 116580. doi: 10.1016/j.jenvman.2022.116580 36323116

[B48] Tett P. (1987). The ecophysiology of exceptional blooms. Rapport Proces-verbaux Des. Reunions. Conseil Int. pour l’Exploration la Mer 187, 47–60.

[B49] Viner-Mozzini Y. Zohary T. Gasith A. (2003). Dinoflagellate bloom development and collapse in Lake Kinneret: A sediment trap study. J. Plankton Res. 25, 591–602. doi: 10.1093/plankt/25.6.591

[B52] Wang L. Cai Q. Xu Y. Kong L. Tan L. Zhang M. (2011). Weekly dynamics of phytoplankton functional groups under high water level fluctuations in a subtropical reservoir-bay. Aquat. Ecol. 45, 197–212. doi: 10.1007/s10452-010-9346-4

[B51] Wang J. Wang B. Luo Z. (1997). Glossary of the yangtze river (Wuhan, China: Wuhan Press).

[B50] Wang H. Zhao D. Chen L. Giesy J. P. Zhang W. Yuan C.. (2020). Light, but not nutrients, drives seasonal congruence of taxonomic and functional diversity of phytoplankton in a eutrophic highland lake in China. Front. Plant Sci. 11. doi: 10.3389/fpls.2020.00179 PMC7067047 32210990

[B53] Wetzel R. Likens G. (2000). Limnological analyses Vol. 3 (Springer: New York, America). doi: 10.1007/978-1-4757-3250-4

[B55] Wu X. Li C. Chen L. Zhao Y. Wang H. (2017). Response mechanism of *Peridiniopsis* bloom to phosphorus in Xiangxi River Bay of Three Gorges Reservoir. J. Lake Sci. 29, 1054–1060. doi: 10.18307/2017.0503

[B54] Wu T. Qin B. Brookes J. Shi K. Zhu G. Zhu M.. (2015). The influence of changes in wind patterns on the areal extension of surface cyanobacterial blooms in a large shallow lake in China. Sci. Total Environ. 518, 24–30. doi: 10.1016/j.scitotenv.2015.02.090 25747360

[B56] Yang Y. Pan J. Y. Han B. P. Naselli-Flores L. (2020). The effects of absolute and relative nutrient concentrations (N/P) on phytoplankton in a subtropical reservoir. Ecol. Indic. 115, 106466. doi: 10.1016/j.ecolind.2020.106466

[B57] Yatigammana S. K. Ileperuma O. A. Perera M. B. U. (2011). Water pollution due to a harmful algal bloom: A preliminary study from two drinking water reservoirs in Kandy, Sri Lanka. J. Natl. Sci. of 39, 91–94. doi: 10.4038/jnsfsr.v39i1.2930

[B58] Zeng C. Xing R. Huang B. (2023). Phytoplankton in headwater streams: spatiotemporal patterns and underlying mechanisms. Front. Plant Sci. 14. doi: 10.3389/fpls.2023.1276289 PMC10628446 37941677

[B59] Zhao W. Li Y. Jiao Y. Zhou B. Vogt R. D. Liu H.. (2017). Spatial and temporal variations in environmental variables in relation to phytoplankton community structure in a eutrophic river-type reservoir. Water 9, 754. doi: 10.3390/w9100754

[B60] Zhou B. Shi K. Wang W. Zhang D. Qin B. Zhang Y.. (2022). Phytoplankton succession phenology trends in the backwaters of the three gorges reservoir in China and their drivers: Results from satellite observations. Ecol. Indic. 143, 109435. doi: 10.1016/j.ecolind.2022.109435

[B61] Zhou G. Zhao X. Bi Y. Liang Y. Hu J. Yang M.. (2011). Phytoplankton variation and its relationship with the environment in Xiangxi Bay in spring after damming of the Three-Gorges, China. Environ. Monit. Assess. 176, 125–141. doi: 10.1007/s10661-010-1571-8 20563898

[B62] Znachor P. Nedoma J. Hejzlar J. Seda J. Komarkova J. Kolař V.. (2020). Changing environmental conditions underpin long-term patterns of phytoplankton in a freshwater reservoir. Sci. Total Environ. 710, 135626. doi: 10.1016/j.scitotenv.2019.135626 31784170

